# Flow Diversion for Intracranial Aneurysms Beyond the Circle of Willis

**DOI:** 10.3389/fneur.2021.674966

**Published:** 2021-05-31

**Authors:** Jinlu Yu, Xianli Lv

**Affiliations:** ^1^Department of Neurosurgery, The First Hospital of Jilin University, Changchun, China; ^2^Department of Neurosurgery, Beijing Tsinghua Changgung Hospital, School of Clinical Medicine, Tsinghua University, Beijing, China

**Keywords:** pipeline embolization device, endovascular treatment, circle of Willis, distal aneurysm, complex aneurysm

## Abstract

**Background:** Few reports have shown the therapeutic outcomes of flow diversion (FD) for intracranial aneurysms beyond the circle of Willis, and the efficacy of this technique remains unclear.

**Materials and methods:** A retrospective study was performed on 22 consecutive patients, diagnosed with intracranial aneurysms beyond the circle of Willis, and treated with pipeline embolization device (PED) (Medtronic, Irvine, California, USA) between January 2015 and December 2019.

**Result:** The 22 patients were between 16 and 66 years old (mean 44.5 ± 12.7 years), and six patients were male (27.3%, 6/22). Twenty-two patients had 23 aneurysms. The 23 aneurysms were 3–25 mm in diameter (12.2 ± 7.1 mm on average). The diameter of the parent artery was 1.3–3.0 mm (2.0 ± 0.6 mm on average). The 23 aneurysms were located as follows: 17 (73.9%, 17/23) were in the anterior circulation, and 6 (26.1%, 6/23) were in the posterior circulation. PED deployment was technically successful in all cases. Two overlapping PEDs were used to cover the aneurysm neck in 3 cases. One PED was used to overlap the two tandem P1 and P2 aneurysms. Other cases were treated with single PED. Coil assistance was used to treat 7 aneurysms, including 4 recurrent aneurysms and 3 new cases requiring coiling assistance during PED deployment. There were no cases of complications during PED deployment. All patients were available at the follow-up (mean, 10.9 ± 11.4 months). All patients presented with a modified Rankin Score (mRS) of 0. During angiographic follow-up, complete embolization was observed in 22 aneurysms in 21 patients, and one patient had subtotal embolization with the prolongation of stasis in the arterial phase.

**Conclusion:** PED deployment for intracranial aneurysms beyond the circle of Willis is feasible and effective, with high rates of aneurysm occlusion.

## Introduction

Currently, flow diversion (FD) has revolutionized the treatment of intracranial aneurysms into a safe and efficacious therapy for large or giant wide-necked aneurysms. However, the off-label uses of FD have increased for intracranial aneurysms, including those in distal locations and bifurcation aneurysms (1).

Currently, FD for aneurysms beyond the circle of Willis is effective, but there are some uncertain factors (2). This is because smaller arteries, the technical challenges of distal navigation, and the coverage of bifurcation branches and perforators may increase the risk of treatment-related complications (3). Thus, these aneurysms remain difficult to treat (4, 5).

Therefore, this study planned to evaluate the safety and efficacy of pipeline embolization device (PED) (Medtronic, Irvine, California, USA) treatment of intracranial aneurysms beyond the circle of Willis, including distal anterior circulation aneurysms and posterior circulation aneurysms.

## Materials and Methods

From January 2015 to December 2019, consecutive 22 patients, who underwent PED treatment for intracranial aneurysms beyond the circle of Willis, were retrospectively reviewed.

### Inclusion Criteria

(1) The location of intracranial aneurysms was beyond the circle of Willis. (2) These aneurysms, including previously coiled aneurysms, underwent treatment with a PED.

### Perioperative Data Collection

The data collected and recorded included age, sex, clinical presentation, aneurysm side, aneurysm size, number of PED deployments, coiling assistance, and procedural complications.

### Scheme of Treatment

#### Medication Management

Dual-antiplatelet medication with aspirin 100 mg and clopidogrel 75 mg was given for at least 5 days before the treatment. In the case of platelet inhibition of 40% to adenosine diphosphate (ADP), an additional 300-mg loading dose of clopidogrel was administered before the procedure. Dual-antiplatelet therapy was maintained for 6 months. Then, aspirin 100 mg was given for a minimum of 6 months or for life.

#### Treatment Procedure

All patients were treated under general anesthesia via a transfemoral approach. A coaxial system consisting of a Shuttle sheath, a guide catheter, an intermediate catheter and a microcatheter was used. Under roadmap guidance, the 0.027-inch Marksman or Phenom catheter (Medtronic, Irvine, California, USA) was navigated beyond the aneurysm neck. Based on the aneurysm neck and parent artery parameters, a PED was chosen to allow enough wall apposition and coverage of the aneurysm. If the aneurysm was ruptured or when necessary, PED deployment plus coiling was performed. Control angiography was performed at 10 and 20 min intervals after PED deployment to observe platelet aggregation within the stent (5).

### Prognostic Evaluation

The modified Rankin Scale (mRS) was used for clinical outcome assessment. During the follow-up imaging, follow-up angiography was analyzed. If the treatment was incomplete, the degree could be evaluated with the prolongation of stasis, which was divided into arterial, capillary, and venous phases.

## Results

### General Information

Twenty-two patients were identified, with ages ranging from 16 to 66 years (mean, 44.5 ± 12.7 years), and six patients were male (27.3%, 6/22). Seventeen patients were admitted for accidental findings, 1 had subarachnoid hemorrhage (SAH), and 4 recurrent aneurysms were treated with previous coiling with or without stenting assistance.

### Imaging Characteristics

Twenty-two patients had 23 aneurysms, of which 12 aneurysms were on the left side and 11 were on the right side. In 23 aneurysms, 2 aneurysms were in tandem. The other 21 patients had single aneurysms. The 23 aneurysms were 3–25 mm in diameter (12.2 ± 7.1 mm on average). The diameter of the parent artery was 1.3–3.0 mm (2.0 ± 0.6 mm on average). The locations of 23 aneurysms were as follows: the first segment of the middle cerebral artery (MCA) (M1), 6 aneurysms; the second segment of the MCA (M2), 2 aneurysms; the third segment of the MCA (M3), 6 aneurysms; the second segment of the anterior cerebral artery (ACA) (A2), 3 aneurysms; the first segment of the posterior cerebral artery (PCA) (P1), 3 aneurysms; the second segment of the PCA (P2), 2 aneurysms; and the third segment of the posterior inferior cerebellar artery (PICA) (p3), 1 aneurysm.

### Treatment Procedure

PED deployment was technically successful in all cases. Two overlapping PEDs were used to cover the aneurysm neck in 3 cases. One PED was used to overlap the tandem P1 and P2 aneurysms. The other cases were treated with single PED. In 23 aneurysms, coiling assistance was performed for 3 aneurysms, including one ruptured aneurysm. In total, coiling was used to treat 7 aneurysms, including 4 recurrent aneurysms and 3 new cases requiring coiling assistance during PED deployment. During PED deployment, the branches were covered by the PED in 15 cases (68.2%, 15/22), according to the results of immediate angiography.

### Follow-Up Outcomes

**Figure 1:**
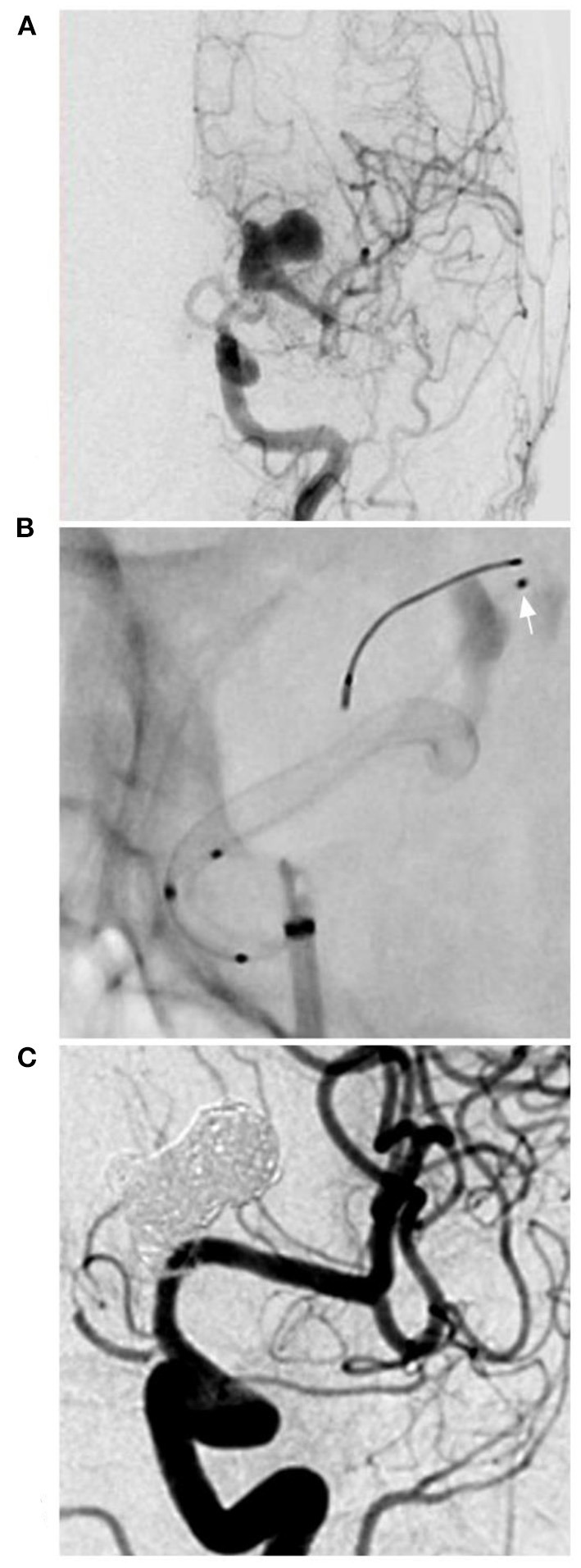
PED for an M1 complex aneurysm. **(A)** DSA of the anterior-posterior view of the ICA showing a complex lobulated aneurysm on the M1 segment of the middle cerebral artery. **(B)** X-ray film showing the deployment of the PED and the microcatheter (arrow) in the aneurysm to plan coiling. **(C)** Follow-up DSA showing complete aneurysm occlusion. DSA, digital subtraction angiography; ICA, internal carotid artery; PED, pipeline endovascular device.

**Figure 2:**
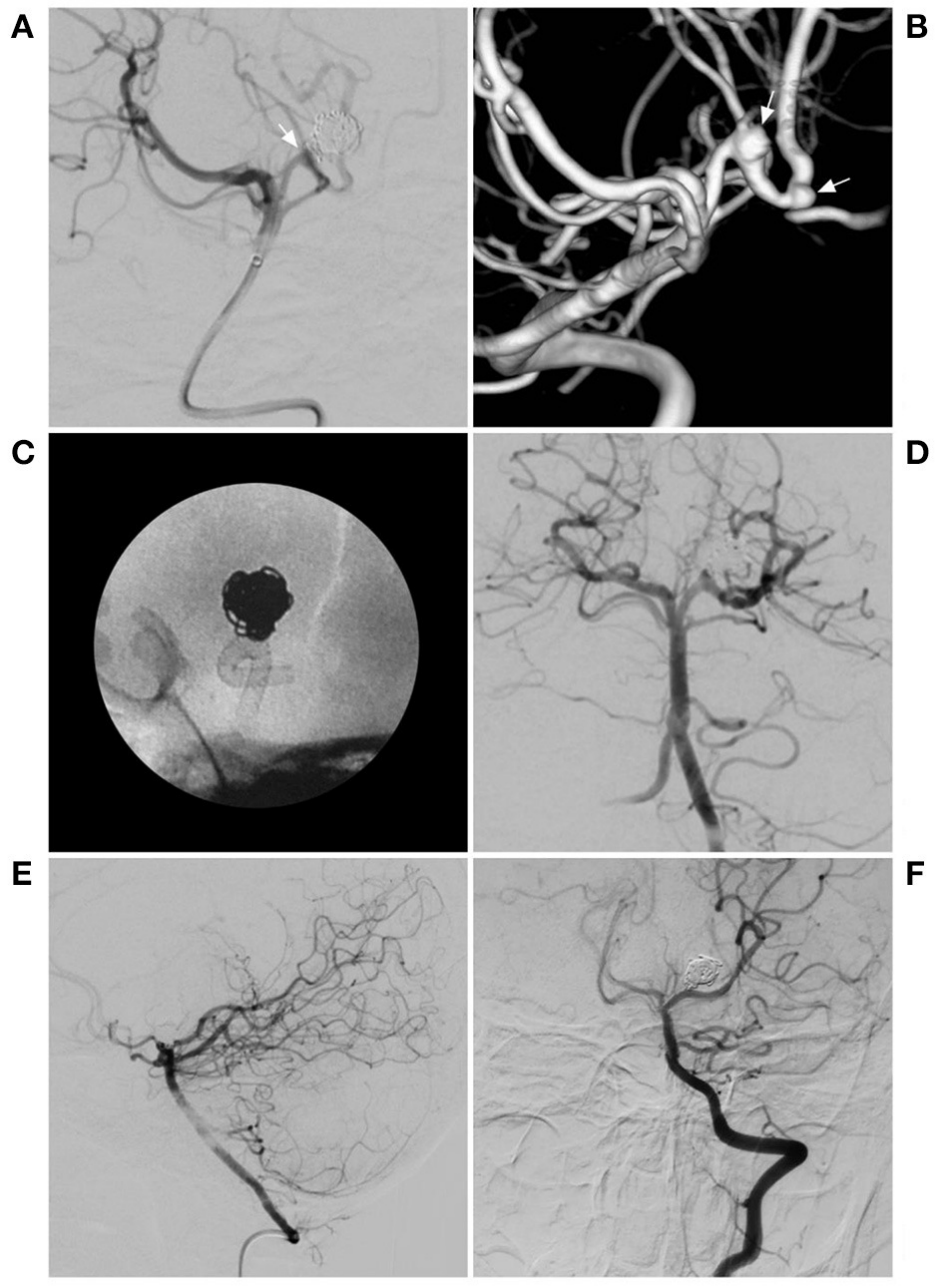
PED for a previously coiled recurrent aneurysm. **(A)** DSA of the BA showing the previous coiled aneurysm in posterior cerebral artery. The arrow indicates the recurrent neck of the aneurysm. **(B)** 3D reconstruction of DSA showing the 2 aneurysms, including the previous coiled aneurysm and another aneurysm (arrows). **(C)** X-ray film showing the deployment of the PED. **(D)** Immediate angiography showing the deployment of the PED. **(E,F)** Follow-up DSA of the VA showing complete aneurysm occlusion. BA, basilar artery; DSA, digital subtraction angiography; PED, pipeline endovascular device; VA, vertebral artery.

All patients were available at the clinical follow-up, and the clinical and imaging follow-up ranged from 3 to 48 months (mean, 10.9 ± 11.4 months). All patients presented with a mRS score of 0 (100%). The degree of embolization was 100% occlusion in 22 aneurysms (95.6%, 22/23), and one aneurysm exhibited <90% occlusion (subtotal embolization with the prolongation of stasis in the arterial phase). Representative cases are shown in Figures 1, 2. Clinical data in this study are summarized in Table 1.

**Table 1:** Clinical data in this study.

**No**.	**Age/sex**	**Onset**	**Side**	**Aneurysm location**	**Parent artery diameter (mm)**	**Size (diameter, mm)**	**Flow diversion**	**Coiling assistance**	**Covered branch**	**Immediate angiography**	**Follow-up time**	**Occlusion (%)**
1	43/M	Accidental	L	M1	3	15	1 Pipeline	N	Lenticular artery	Incomplete	37 mon	100
2	59/F	Recurrent	R	M1	2.7	20	1 Pipeline	N	Lenticular artery	Incomplete	7 mon	100
3	16/F	Recurrent	R	M2	2	10	1 Pipeline	N	Lenticular artery, superior trunk	Incomplete	24 mon	100
4	66/M	Accidental	R	M2	2.2	7	1 Pipeline	N	Lenticular artery, superior trunk	Incomplete	6 mon	<90
5	36/F	Accidental	R	M3	1.7	8	1 Pipeline	N	No	Incomplete	6 mon	100
6	55/F	Accidental	L	M3	1.5	8	1 Pipeline	N	No	Incomplete	48 mon	100
7	57/F	Accidental	R	M3	1.8	9	2 Pipelines	N	No	Incomplete	6 mon	100
8	50/F	Accidental	L	M1	3	13	2 Pipelines	N	Lenticular artery	Incomplete	8 mon	100
9	54/F	SAH	R	M1	2.8	25	1 Pipeline	Y	Lenticular artery	Incomplete	3 mon	100
10	60/F	Accidental	L	P1	1.5	7	1 Pipeline	N	Perforating artery	Incomplete	11 mon	100
11	23/F	Recurrent	L	P1	2	3	1 Pipeline	N	Perforating artery	Incomplete	18 mon	100
12	54/M	Accidental	L	P1 and P2	2	3 and 4	1 Pipeline	N	Perforating artery	Incomplete	6 mon	100
13	37/M	Accidental	L	M1	2.9	25	1 Pipeline	Y	Lenticular artery	Incomplete	6 mon	100
14	45/M	Accidental	L	M1	2.7	10	1 Pipeline	N	Lenticular artery	Incomplete	6 mon	100
15	46/F	Accidental	L	M3	2	12	1 Pipeline	N	No	Incomplete	7 mon	100
16	49/F	Accidental	R	M3	1.9	20	1 Pipeline	N	No	Incomplete	8 mon	100
17	28/F	Accidental	R	A2	1.5	20	1 Pipeline	N	Pericallosal artery	Incomplete	3 mon	100
18	40/M	Accidental	L	A2	1.6	5	1 Pipeline	N	Pericallosal artery	Incomplete	6 mon	100
19	30/F	Accidental	R	A2	1.4	20	1 Pipeline	Y	Pericallosal artery	Incomplete	6 mon	100
20	37/F	Accidental	L	M3	1.5	20	2 Pipelines	N	No	Incomplete	6 mon	100
21	48/F	Accidental	R	P2	1.3	12	1 Pipeline	N	Perforating artery	Incomplete	6 mon	100
22	46/F	Recurrent	R	p3	1.5	6	1 Pipeline	N	No	Incomplete	6 mon	100

*A2, anterior cerebral artery (2 was the second segment); DSA, digital subtraction angiography; F, female, L, left, M, male, M1-3, middle cerebral artery (1–3 was the first-third segment); Mon, month; N, no; P1-2, posterior cerebral artery (1–2 was the first-second segment); p3, posterior inferior cerebellar artery (3 was the third segment); R, right; SAH, subarachnoid hemorrhage; Y, yes*.

*Note, in the onset column, “recurrent” refers to previous coiling*.

## Discussion

FD involves 24–55% metal coverage, and after FD deployment, the blood flow within the aneurysm is disturbed, causing stasis that leads to thrombosis, followed by endothelialization of the parent artery (6). Currently, FD technology has revolutionized the treatment of intracranial aneurysms that are suboptimal for surgical or traditional interventional treatment (7). For aneurysms beyond the circle of Willis, classic endovascular approaches to the treatment of these aneurysms include selective coiling or parent artery occlusion, which imparts risks of recurrence and distal infarction (8).

The Pipeline for Uncoilable or Failed Aneurysms (PUFS) trial showed the safety and effectiveness of the use of PEDs in the treatment of large and giant wide-neck aneurysms of the internal carotid artery in adult patients (9). At the same time, based on their ability to reconstruct the parent artery, the off-label uses of FD are constantly extended, including aneurysms beyond the circle of Willis (1). These aneurysms are often dissected and located in sub-2.0-mm vessels, where small-diameter PEDs have been used (5). In this study, we also tried to treat 22 patients with 23 aneurysms with the deployment of PEDs.

The deployment of FDs in arteries beyond the circle of Willis is technically challenging due to the smaller caliber of the parent vessel and the relative stiffness of the high-metal coverage stent. Sometimes, telescoping PEDs with 25–30% overlap is a feasible low-risk treatment option for long-segment aneurysms, using larger-diameter PEDs more proximally (10).

FD among aneurysms beyond the circle of Willis is effective (5). In the Ravindran et al. study of the use of FD for distal circulation aneurysms, complete and near-complete occlusion was noted in 78.2% of aneurysms (11). Our study demonstrates that PED treatment for aneurysms beyond the circle of Willis is effective, with rates of complete occlusion close to 95.6%.

FD can be applied alone or in combination with coiling, which includes the retreatment of previously coiled lesions, theoretically, which allows higher rates of occlusion than treatment with FDs alone, such as the case shown in Figure 2 (11). However, coiling assistance is controversial, especially for large and giant aneurysms, and despite coiling assistance in FD deployment, delayed rupture cannot completely be avoided. Moreover, after coiling assistance, the effect may not be complete, and the coiling could result in the occlusion of a perforating artery.

Intracranial aneurysms beyond the circle of Willis are often dissecting and long but not large. Delayed rupture was uncommon after FD deployment, so the aim of coiling assistance was not to reduce the rupture risk; coiling may increase the degree of aneurysm occlusion. The coiling assistance during FD deployment was the same as that during conventional stent-assisted coiling. For instance, in the case shown in Figure 1, follow-up showed excellent occlusion after coiling assistance. However, coiling assistance is selectively applied for intracranial aneurysms beyond the circle of Willis, because in these aneurysms, the blood flow is not abundant, and FDs alone may be sufficient in most of these cases. In our study, 7 aneurysms were treated with coils in the aneurysms, including 4 recurrent aneurysms and 3 new cases requiring coiling assistance during PED deployment, and complete occlusion was obtained. Coiling assistance was feasible, but whether there is a difference between aneurysms with or without previously coiling requires further study. In our study, due to the small number of cases, it was difficult to identify such a difference. However, PED deployment was safe and effective.

However, the complications associated with FD deployment are not negligible and include ischemic/thromboembolic and hemorrhagic complications (9, 12). In addition, for small vessels, after FD deployment, segmental vasospasm can occur as a frequent vascular reaction, potentially causing symptomatic ischemia or even stroke ~1 month after the procedure (13). Safety concerns regarding FD within small vessels can originate with vessel trauma from robust support to deliver and open the PED in the distal circulation, often in the presence of significant tortuosity, acute stent thrombosis, and delayed in-stent stenosis (5, 14).

The ASPIRe (Aneurysm Study of Pipeline in an Observational Registry) meta-analysis reported outcomes with a major morbidity of 6.8% and mortality of 1.6% across on-label PED treatments (15). In the Bender et al. study of FD for aneurysms in distal vessels measuring <2.0 mm, the major morbidity of 4.5% and mortality of 1.5% observed were lower than the on-label PED series outcomes (5). In the Primiani et al. report of A2, M2, and P2 aneurysms and beyond, the procedural compilation rate of 7.7% indicates a need for further studies as flow diversion technology constantly evolves (16). Our study reported no complications because the choice of cases was appropriate.

To reduce ischemic complications, instead of a PED with 30% metal coverage, an intermediate-porosity braided LEO stent (Balt Extrusion, Montmorency, France) with 14% metal coverage can be used with the help of a flow-diversion effect (17). In the Cagnazzo et al. study of 76 intracranial aneurysms and 98 side branches covered by LEO stents, the rate of flow remodeling on the covered arteries and perforators was 9 and 4%, respectively, and complete occlusion of aneurysms treated with sole stent-placement therapy was 70% (18).

In addition, a new low-profile visualized intraluminal support device (LVIS Blue; MicroVention, Tustin, California, USA) is a braided stent that provides a higher degree of metal coverage (22–28%) than first-generation devices (19). Although the coverage of the LVIS Blue stent is lower than that of FDS, the LVIS Blue stent may be beneficial for complete obliteration of an aneurysm due to not only its support of a high occlusion rate using coils inside of the aneurysm but also its flow-diversion effect (20, 21).

## Conclusions

The PED is an effective tool for managing aneurysms beyond the circle of Willis, especially those that are difficult to reconstruct with clipping and residual or recanalizing aneurysms after coiling.

## Data Availability Statement

The raw data supporting the conclusions of this article will be made available by the authors, without undue reservation.

## Ethics Statement

Ethical review and approval was not required for the study on human participants in accordance with the local legislation and institutional requirements. Written informed consent for participation was not required for this study in accordance with the national legislation and the institutional requirements.

## Author Contributions

JY: draft. XL: review, editing, and submitting. All authors read and approved the final version of the manuscript.

## Conflict of Interest

The authors declare that the research was conducted in the absence of any commercial or financial relationships that could be construed as a potential conflict of interest.
